# Editorial: Cognitive and mental health improvement under- and post-COVID-19

**DOI:** 10.3389/fpsyg.2025.1565941

**Published:** 2025-03-06

**Authors:** Chong Chen, Gabriele Nibbio, Yuka Kotozaki

**Affiliations:** ^1^Division of Neuropsychiatry, Department of Neuroscience, Yamaguchi University Graduate School of Medicine, Ube, Yamaguchi, Japan; ^2^Department of Clinical and Experimental Sciences, University of Brescia, Brescia, Italy; ^3^Department of Hygiene and Preventive Medicine, School of Medicine, Iwate Medical University, Morioka, Iwate, Japan

**Keywords:** emotional wellbeing, cognitive function, resilience, posttraumatic growth, anxiety, mindfulness, gender difference, social support

## Introduction

An increasing body of research suggests that the COVID-19 pandemic has exacerbated mental health challenges worldwide (Santomauro et al., 2021; Penninx et al., 2022; Chen et al., 2023; Barlati et al., 2021; Harrison and Taquet, 2023), including higher levels of stress, depression, anxiety, and suicidal ideation, as well as a notable rise in cognitive impairments (Harrison and Taquet, 2023; Corbett et al., 2023; Galderisi et al., 2024; Nibbio et al., 2025). While the direct biological effects of the virus and the psychological toll of infection have contributed to these outcomes (Penninx et al., 2022; Harrison and Taquet, 2023), growing evidence underscores that key drivers also include a pervasive fear of contagion (Alimoradi et al., 2022; Chen et al., 2024a) and prolonged feelings of loneliness and social isolation (Hagiwara et al., 2024; Chen et al., 2024b). These psychosocial stressors have affected individuals across various age groups and socioeconomic backgrounds.

Although the World Health Organization officially declared an end to COVID-19 as a global public health emergency on May 5, 2023—after initially proclaiming it on January 30, 2020—new infections continue to be reported around the world. Moreover, the psychological toll remains evident: studies indicate that loneliness, which surged due to stringent quarantine and physical-distancing measures, has yet to return to pre-pandemic levels (Kullgren et al., 2023; Sugaya et al., 2024). Many individuals continue to experience a sense of isolation, reflecting the deep social and emotional impact of pandemic restrictions.

These developments emphasize the urgent need for initiatives to enhance cognitive and mental wellbeing during and beyond the pandemic era. By addressing persistent challenges, health professionals, policymakers, and communities can work together to foster resilience, reduce long-term psychological harm, and support cognitive recovery.

This Research Topic compiles 23 studies aimed at addressing these unresolved issues and advancing understanding of interventions that promote cognition and mental health. The participants represent diverse populations, including COVID-19 patients (Bonfim et al.; Li et al.), healthcare professionals (Cheng et al.; Jiang et al.; de Vroege and van den Broek; Zeng et al.; Zhu et al.), medical students (Wang, Zhang, et al.), the general population (Barbalat et al.; Hao et al.; Kulbin et al.; Sasaki et al.), college students (Qing et al.; Shi; Tang and He), and school teachers (Vega-Fernández et al.).

The topics of these studies are equally diverse. They explore mental health during and after the pandemic, identify risk and protective factors—such as emotion malleability beliefs (Sasaki et al.), self-control (He et al.), social support (Shi), and active leisure engagement (Kulbin et al.)—and evaluate the effects of various interventions. Interventions studied include exercise (Wang, Tian, et al.), exposure to urban green spaces (Patwary et al.), online mindfulness programs (Melvin et al.), multicomponent lifestyle medicine (Wong et al.), and light therapy (Chen et al.).

## Cognitive and mental health status

Being infected by COVID-19 represents a major psychological stressor, with the virus itself exerting direct biological effects that may contribute to cognitive and mental health challenges. In a retrospective study conducted in Brazil, Bonfim et al. reported that 24% of 630 outpatients, confirmed COVID-19 positive between December 2020 and March 2022, exhibited cognitive symptoms. These symptoms ranged from difficulties with attention and memory to impaired thinking. Depression, fatigue, female gender were identified as risk factors for these cognitive symptoms. Similarly, Li et al. surveying 482 patients in China from December 2022 to June 2023, found that 13% experienced symptoms of depression, 27% anxiety, and 25% stress. The severity of long-term COVID symptoms correlated with poorer mental health outcomes, while resilience and social support emerged as protective factors.

While infection-control measures have been effective in limiting the spread of the virus, they have often come at a cost to mental health. For example, Barbalat et al. conducted a nationwide online survey during France's first lockdown (March–May 2020), involving approximately 19,000 participants. They observed a gradual decline in mental health over the course of the lockdown, with psychiatric conditions and concerns about access to protective equipment serving as risk factors. Conversely, optimism about the pandemic's trajectory, neighborhood support, and participation in collective actions were protective factors. Similarly, Qing et al. examined the impact of campus lockdowns on Chinese university students and found elevated levels of stress and depression compared to pre-lockdown periods. Using latent class mixed models, the authors identified distinct mental health trajectories and found that students with unfavorable peer relationships were more likely to experience poorer mental health trajectories.

Healthcare professionals, frequently exposed to patients and heavy workloads, have reported high levels of stress and mental health challenges during the pandemic (Hacimusalar et al., 2020). Zhu et al. surveyed 145 members of the medical security team at the 2022 Winter Olympic Games and Paralympics and found that longer work durations were associated with worse mental health outcomes, including higher levels of depression and anxiety. Females were particularly vulnerable. Such findings underscore the unique pressures faced by frontline workers during public health crises.

The pandemic's impact extends beyond mental health to physical wellbeing and motivation. Vega-Fernández et al., in a study of 161 Chilean school teachers conducted in late 2021, reported that 98% experienced musculoskeletal disorders in the past year. These disorders were associated with poorer physical and mental quality of life. In this study, females were also more likely to have musculoskeletal disorders. Tang and He found that college students with high depressive symptoms during the pandemic showed reduced academic engagement, highlighting the broader motivational consequences of mental health struggles.

As COVID-19 symptoms have become milder and restrictions relaxed, new mental health challenges have emerged. Hao et al. reported that after China eased its COVID-19 control measures in January 2023, about one in five individuals exhibited over-concern about the virus, characterized by obsessive thoughts and anxiety regarding infection. Poor self-rated health and worries about family members contracting the virus were associated with these over-concerns. Cheng et al., in a survey of over 2,000 healthcare professionals shortly after these relaxations, found that over half reported symptoms of depression and anxiety, with female gender, younger age, low professional rank, and longer working hours identified as risk factors. In another survey of healthcare professionals, Jiang et al. further suggested reciprocal relationships between mental health problems and job burnout during this period.

Despite the general decrease in infection risk and social isolation as the pandemic winds down, studies indicate that loneliness, heightened by stringent quarantine measures, has not fully returned to pre-pandemic levels (Kullgren et al., 2023; Sugaya et al., 2024). de Vroege and van den Broek surveyed 510 mental healthcare professionals and found improvements in work-life balance and reductions in mental health complaints post-pandemic compared to earlier stages. However, 36% reported increased stress and 21% experienced more depression. Wang, Zhang, et al. also noted that one in four medical students in March 2023 reported high stress levels. These findings emphasize the importance of ongoing mental health monitoring and tailored care, even as the pandemic subsides.

Not all outcomes of the pandemic have been negative. Some individuals have demonstrated resilience and even posttraumatic growth, characterized by deeper appreciation for life and more meaningful interpersonal relationships. For example, Zeng et al. reported that 39% of Chinese resident physicians surveyed in March 2023 exhibited posttraumatic growth. Satisfaction with income and sufficient workplace support were key factors associated with posttraumatic growth. These findings highlight the potential to foster resilience and posttraumatic growth and offer a pathway to improved mental health outcomes.

## Risk and protective factors

### Female gender

A consistent risk factor identified in the above mentioned studies is being female (Bonfim et al.; Zhu et al.; Vega-Fernández et al.; Cheng et al.). This gender difference is not a new finding; epidemiological studies have long observed that females are at higher risk of developing psychiatric disorders, particularly depressive (Salk et al., 2017) and anxiety disorders (McLean et al., 2011). Several mechanisms have been proposed to explain this vulnerability. For instance, females have been reported to experience more severe forms of adverse childhood experiences, predisposing them to mental health challenges (Hirai et al., 2025). Moreover, females are more likely to engage in rumination (i.e., repetitive and passive focus on negative emotional experiences) (Johnson and Whisman, 2013) and exhibit risk-aversive behaviors in high stress situations (Lei et al., 2021). They are also less likely to engage in intense physical activity (Nakagawa et al., 2020), which has been associated with mental health benefits (Chen and Nakagawa, 2023a,b). Another explanation is highlighted by Sasaki et al., who investigated the role of emotion malleability beliefs. Their findings suggest that females are more likely to hold fixed beliefs that they cannot control or change their emotions. These beliefs were linked to higher levels of psychological distress and were particularly prevalent among women, in those under 45 years old, and those with psychiatric disorders.

### Social support

Social support, consistent with extensive evidence (Taylor, 2011; Chen, 2017a), has been identified as a crucial protective factor. In addition to the findings by Li et al., Barbalat et al., Qing et al., and Zeng et al., Shi reported that college students with higher levels of social support experienced lower levels of depression and anxiety. The study suggested that social support may enhance perceived control, while a lack of support could lead to a sense of helplessness, a key factor contribute to the development of depressive states (Maier and Seligman, 2016). Perceived control promotes problem-focused coping strategies that help address stressors, while low perceived control is associated with maladaptive coping strategies, such as avoidance, rumination, and suppression, which increase the risk of psychopathology (Lincoln et al., 2022).

### Self-control

Self-control also appeared as an important protective factor. He et al. found a negative correlation between self-control and symptoms of depression, anxiety, and irritability in college students. Using network analysis, they identified impulse control as a bridge between self-control and irritability or anxiety symptoms, while resistance to temptation acted as a bridge between self-control and depressive symptoms. Self-control, which enables individuals to manage their emotions, behaviors, and thoughts, is a crucial determinant of wellbeing (Tangney et al., 2018), including emotional development (Eisenberg et al., 2014).

### Leisure engagement

In addition to social support and self-control, active leisure engagement has emerged as another key protective factor against stress and mental health challenges. Kulbin et al., using latent profile analysis, categorized 439 Estonia adults into four distinct trajectories based on changes in stress and coping. They found that participants in the healthiest trajectories reported higher levels of active leisure engagement, such as physical exercise, spending time in nature, and pursuing hobbies. These activities are known to promote mental health and resilience (see also the next section) (Taquet et al., 2016; Chen, 2017b; Koga et al., 2023).

Together, these findings highlight the importance of a multifaceted approach to mental wellbeing, combining psychological, behavioral, and social strategies to mitigate the risk of psychopathology.

## Interventional strategies

The current Research Topic offers valuable insights into the benefits of various intervention strategies for mental health.

### Physical exercise

Wang, Tian, et al. conducted a systematic review and meta-analysis to assess the effects of physical exercise interventions on mental health. Based on 12 identified studies, they estimated the effect size as follows: for depression, standardized mean difference (SMD) = −1.02 (95% CI: −1.42 to −0.62); for anxiety, SMD = −0.81 (95% CI: −1.10 to −0.52); for stress, SMD = −1.05 (95% CI: −1.33 to −0.78). The greatest benefits were observed with single exercise sessions lasting 30–40 min and a frequency of 3–5 times per week. These findings align with extensive evidence supporting the mental health benefits of regular physical activity (Nakagawa et al., 2020; Chen and Nakagawa, 2023a,b; Chen, 2017b; Koga et al., 2023; Sakai et al., 2021; Deste et al., 2023; Chen et al., 2017). The biological mechanisms behind these effects include the release of neurotrophic factors, endorphins, and endocannabinoids, along with activation of the dopamine and serotonin neurotransmitter systems (Chen and Nakagawa, 2023a,b; Chen et al., 2017, 2016; Hou et al., 2024).

### Nature contact

Patwary et al. found that spending more time outdoors in green spaces after lockdowns significantly improved mental health, reducing symptoms of depression and anxiety. This finding is consistent with the growing interest in nature and horticultural therapy (Chen, 2018a; Chen and Nakagawa, 2018, 2019; Yamashita et al., 2021; Mizumoto et al., 2024). The benefits of nature contact are linked to the relaxation of the brain, as well as endocrine and immune effects (Chen, 2018a; Chen and Nakagawa, 2018, 2019; Yamashita et al., 2021; Mizumoto et al., 2024). Furthermore, engaging in physical activity while in nature promotes social interactions, reducing loneliness and enhancing perceived social support (Kabisch et al., 2017).

### Mindfulness

Melvin et al. conducted a qualitative, interpretative phenomenological analysis of participants and facilitators in an online mindfulness program during the pandemic. Mindfulness practices, such as meditation and yoga, have been shown to alleviate stress and promote mental health by enhancing body awareness, attention, and emotion regulation, as well as facilitating perspective shifts and clarifying values (Hölzel et al., 2011; Gu et al., 2015). This study identified both the benefits and challenges of delivering mindfulness programs online, providing valuable insights for the future development of more effective programs.

### Multicomponent lifestyle medicine

Wong et al. evaluated an 8-week smartphone-delivered multicomponent lifestyle medicine intervention through a randomized controlled trial in a non-clinical sample. This intervention combined various lifestyle changes, including physical exercise, healthy eating, and mindfulness practices like yoga. While no studies in this Research Topic focused solely on nutrition, evidence supports the critical role of nutrition in proper brain function and mental health (Chen, 2018b; Chen and Nakagawa, 2020). The combination of healthy eating, exercise, and mindfulness is expected to have synergistic effects. Wong et al. reported improvements in overall mental health, including reductions in depressive symptoms, anxiety, stress, and insomnia, with effect sizes ranging from 0.13 to 0.56 (Cohen's d). Notably, the benefits persisted at a 1-month follow-up.

### Light therapy

Chen et al. conducted a systematic review and meta-analysis on the effects of light therapy for depression in adolescents and young adults. The estimated effect sizes were: SMD = −2.1 (95% CI: −2.5 to −1.68) for individuals using concurrent medications, and SMD = −1.03 (95% CI: −1.27 to −0.78) for individuals without concurrent medications. Light therapy is thought to reset altered circadian and seasonal rhythms in depressive disorders (Pail et al., 2011). The authors also performed additional analyses to determine optimal dosing guidelines, providing insights for clinical applications.

## Concluding remarks

We hope this Research Topic has offered valuable perspectives on the mental health challenges exacerbated by the COVID-19 pandemic and the strategies to address them. The findings highlight the intricate interplay of psychological, behavioral, and social factors in shaping mental health outcomes. This calls for a holistic approach that combines individual practices like mindfulness and exercise with broader systemic changes, including improved access to care and strengthened community support. By addressing these issues with a collaborative and interdisciplinary approach, we can transform the lessons of the pandemic into meaningful actions that improve mental health outcomes for future generations. We hope this Research Topic inspires ongoing research, innovation, and policy development to meet these ongoing challenges.
